# Fecal Microbiota Transplant Mitigates Adverse Outcomes Seen in Patients Colonized With Multidrug-Resistant Organisms Undergoing Allogeneic Hematopoietic Cell Transplantation

**DOI:** 10.3389/fcimb.2021.684659

**Published:** 2021-08-27

**Authors:** Andrew J. Innes, Benjamin H. Mullish, Rohma Ghani, Richard M. Szydlo, Jane F. Apperley, Eduardo Olavarria, Renuka Palanicawandar, Edward J. Kanfer, Dragana Milojkovic, Julie A. K. McDonald, Eimear T. Brannigan, Mark R. Thursz, Horace R. T. Williams, Frances J. Davies, Julian R. Marchesi, Jiří Pavlů

**Affiliations:** ^1^Centre for Haematology, Imperial College London at Hammersmith Hospital, London, United Kingdom; ^2^Division of Digestive Diseases, Department of Metabolism, Digestion and Reproduction, Imperial College London, London, United Kingdom; ^3^Medical Research Council (MRC) Centre for Molecular Bacteriology and Infection, Imperial College London, London, United Kingdom; ^4^National Institute for Health Research Health Protection Research Unit in Healthcare Associated Infections and Antimicrobial Resistance, Imperial College London, London, United Kingdom

**Keywords:** antimicrobial resistance, multidrug resistant bacteria, gut microbiota, fecal microbiota transplant, hematopoietic (Stem) cell transplantation (HCT), hematological malignances

## Abstract

The gut microbiome can be adversely affected by chemotherapy and antibiotics prior to hematopoietic cell transplantation (HCT). This affects graft success and increases susceptibility to multidrug-resistant organism (MDRO) colonization and infection. We performed an initial retrospective analysis of our use of fecal microbiota transplantation (FMT) from healthy donors as therapy for MDRO-colonized patients with hematological malignancy. FMT was performed on eight MDRO-colonized patients pre-HCT (FMT-MDRO group), and outcomes compared with 11 MDRO colonized HCT patients from the same period. At 12 months, survival was significantly higher in the FMT-MDRO group (70% *versus* 36% *p* = 0.044). Post-HCT, fewer FMT-MDRO patients required intensive care (0% *versus* 46%, *P* = 0.045) or experienced fever (0.29 *versus* 0.11 days, *P* = 0.027). Intestinal MDRO decolonization occurred in 25% of FMT-MDRO patients *versus* 11% non-FMT MDRO patients. Despite the significant differences and statistically comparable patient/transplant characteristics, as the sample size was small, a matched-pair analysis between both groups to non-MDRO colonized control cohorts (2:1 matching) was performed. At 12 months, the MDRO group who did not have an FMT had significantly lower survival (36.4% *versus* 61.9% respectively, *p*=0.012), and higher non relapse mortality (NRM; 60.2% *versus* 16.7% respectively, *p*=0.009) than their paired non-MDRO-colonized cohort. Conversely, there was no difference in survival (70% *versus* 43.4%, *p*=0.14) or NRM (12.5% *versus* 31.2% respectively, *p*=0.24) between the FMT-MDRO group and their paired non-MDRO cohort. Collectively, these data suggest that negative clinical outcomes, including mortality associated with MDRO colonization, may be ameliorated by pre-HCT FMT, even in the absence of intestinal MDRO decolonization. Further work is needed to explore this observed benefit.

## Introduction

There is mounting evidence that the gut microbiome directly impacts upon immune responses in patients with hematological malignancies (Schluter et al., 2020), and markedly influences clinical outcomes after hematopoietic cell transplantation (HCT) (Peled et al., 2020). In particular, studies to date have shown a relationship between an increased diversity of the gut microbiota at neutrophil engraftment and better long-term survival (Peled et al., 2017; Andermann et al., 2018; Peled et al., 2020), and additionally a higher diversity of the intestinal microbiota is seen to be associated with lower graft *versus* host disease (GvHD)-related mortality (Malard et al., 2018). However, the functionality and composition of the microbiome of HCT patients is profoundly impacted by preceding courses of intensive chemotherapy, as well as the necessary use of antibiotics during those periods to treat febrile neutropenic episodes (Wong and Santiago, 2017). Furthermore, the pre-transplant gut microbiota is predictive of bloodstream infections during transplant; poorer outcomes are seen in patients who have intestinal colonization with multidrug-resistant organisms (MDROs) (Samet et al., 2013), particularly in terms of increased risk of multidrug-resistant bloodstream infection (BSI) (Cattaneo et al., 2018). Higher mortality from invasive MDRO infection is in part a result of the delayed delivery of optimal antimicrobials, as well as the significant toxicity profiles of the regimens (Bilinski et al., 2016; Satlin et al., 2016). Whilst pre-HCT screening for MDRO carriage may help empiric antimicrobial choice, the mortality of MDRO infection remains high (Cattaneo et al., 2018). Biological approaches that may restore the pre-HCT gut microbiota may be beneficial both from a hematological perspective, and for their potential in reducing MDRO titers.

One such potential approach is fecal microbiota transplantation [FMT; also referred to as ‘intestinal microbiome transplantation (IMT)’ (Craven et al., 2020)], which involves the delivery of processed stool derived from healthy, screened donors into the gut of affected patients. When stool was collected from patients with hematological disease prior to antibiotics/HCT and then administered back to them afterwards *via* ‘auto FMT’, gut microbiota diversity and composition was restored back to a pre-morbid pattern (Taur et al., 2018). A more recent study including hematological malignancy patients has helped to further delineate the close interaction between the gut microbiota and neutrophil, lymphocyte and monocyte cell dynamics (Schluter et al., 2020). In addition, FMT is an established treatment for recurrent *Clostridioides difficile* infection (CDI) (van Nood et al., 2013; Mullish et al., 2018), which is also a pathobiont that colonizes the gut microbiome (Relman and Lipsitch, 2018) and has been shown to reduce incidence of BSIs in patients with recurrent CDI (Ianiro et al., 2019). As such, there has been a logical expansion to evaluate the application of FMT for pre-HCT patients where there is a recognized impact of the gut microbiota on outcomes, including gut GvHD (van Lier et al., 2020) and MDRO BSIs (Bilinski et al., 2017; Saha et al., 2019).

In our recent observational study, we reported improved outcomes post FMT from an infection perspective in immunocompromised patients (including patients with hematological malignancy, and those with renal transplantation) colonized with MDROs (Ghani et al., 2020; Mullish et al., 2020), including reduced use of carbapenems and fewer bacteremias. In this study, *via* an initial retrospective analysis and subsequent case-control cohort study, we further evaluate the impact of FMT upon those MDRO-colonized patients with hematological malignancy, with regards to clinically-relevant hematological outcomes, including survival.

## Methods

### Study Design – Overall Approach and Retrospective Analysis

This study was performed in a single center with approximately 1500 inpatient beds. We have been routinely screening all HCT recipients for MDRO prior to admission for HCT *via* rectal swab or stool screening *via* culture or PCR since 2015, and also during the course of their admission (Otter et al., 2020). The aim of the initial retrospective study presented here was to evaluate early outcomes of HCT in patients who underwent FMT, following on from our earlier study looking at outcomes in MDRO-colonized patients. This study was not randomized and entry into the FMT study was offered to selected MDRO-colonized HCT patients since 2016. MDRO-colonized patients who did not receive HCT before 2016 and patients who elected not to undergo FMT were selected as controls (see *Statistical Methods*).

The study was approved by a UK Research Ethics Committee (REC reference: 19/LO/0112). All patients, including patients used as controls, provided informed consent authorizing the use of their personal information for research purposes. MDRO was defined as extended-spectrum beta-lactamase-producing Enterobacteriaceae, vancomycin-resistant enterococci, or carbapenem-resistant Enterobacteriaceae.

### Fecal Microbiota Transplants

In patients with MDRO intestinal colonization (detected on rectal screening) during previous chemotherapy who were scheduled for HCT. Antibiotics were not given to patients as selective digestive decontamination of their colonizing MDRO. FMT was planned to be performed 2-6 weeks before initiation of the transplant conditioning regimen. At the time of FMT, patients had to be clinically infection-free and off antibiotic therapy.

CMV IgG negative donors were used to prevent CMV reactivation/disease (Cheng et al., 2019). In accordance with current UK guidance, all donor products were screened for carbapenemase-producing Enterobacteriaceae, vancomycin-resistant enterococci and extended spectrum beta-lactamase-producing bacteria, to minimize the risk of introducing a new MDRO infection (DeFilipp et al., 2019). The selection of donors and administration of FMT were in accordance with the joint British Society of Gastroenterology (BSG) and Healthcare Infection Society (HIS) guidelines (Mullish et al., 2018); the protocol used was as adapted from our established practice for use of FMT for the treatment of recurrent *C. difficile* infection. FMT recipients all received bowel purgatives on the day prior to the procedure, using a polyethylene glycol (PEG)-based preparation. Patients were administered oral proton pump inhibitor on the night prior to and the morning of the procedure and received metoclopramide approximately 30 minutes prior to FMT administration. Nasogastric tube insertion was performed either on the night before or morning of the procedure, with positioning confirmed using chest radiograph. The FMT used in all procedures had been previously prepared in house anaerobically and stored at -80°C for less than six months, using 10% w/v glycerol as cryopreservative (Mullish et al., 2015; Cammarota et al., 2019). Each FMT unit had been prepared from at least 50 grams of crude stool from a single unrelated healthy screened donor (FMT prepared from five different such donors was used in this study). FMT was thawed on the morning of the procedure at room temperature; when thawed, FMT was diluted to 100ml, and drawn into syringes within an anaerobic cabinet ready for administration. All FMTs were administered prior to the start of the COVID-19 pandemic.

### Statistical Methods and Matched Pair Study

The primary endpoint was survival, with non-relapse mortality (NRM) being a secondary endpoint. Probabilities of survival were calculated using the Kaplan–Meier method, with the log-rank test utilized for comparison of groups; probabilities of NRM were calculated using the cumulative incidence procedure, with disease progression being the competing risk. Gray’s test was used to compare groups. Days of fever were normalized for days of hospital admission, and compared by unpaired, non-parametric testing (Mann–Whitney U-test).

Patient and transplant characteristics were compared using Fisher’s exact test and the Mann-Whitney U-test as appropriate. Statistically, whilst there were no differences in many key clinical variables between the MDRO-colonized patients who underwent FMT (‘FMT MDRO group’) and MDRO-colonized patients who did not have FMT (‘No FMT MDRO group’, **Table 1**), two case-control cohort studies were also conducted (in addition to the direct comparison of these groups) to minimize the effects of known covariates on survival. Specifically, using a 2:1 matched pair analysis to account for the small sample size, we compared outcomes of both the FMT MDRO and No FMT MDRO groups to their respective control cohorts who were not MDRO-colonized, but were matched for disease type, disease stage, transplant intensity, donor type (matched sibling, matched unrelated, and haploidentical), and age. Statistical analyses were undertaken using SPSS v25, and *P*-values <0.05 were taken as statistically significant.

**Table 1:** Demographics and further clinical details of MDRO-colonized HCT recipients.

Characteristic	FMT MDRO (n = 8)	No FMT MDRO (n = 11)	*P*
**Patient age (years)**			
** (Median, range)**	61.9 (33-70)	59.8 (31-66)	0.32
**Diagnosis to HCT (years)**			
** (Median, range)**	0.8 (0.3-9.6)	0.5 (0.3-13.3)	0.89
**Disease**			
** CML**	2 (25%)	2 (18%)	0.68
** AML/MDS**	3 (38%)	7 (64%)	
** ALL**	2 (25%)	1 (9%)	
** T-cell lymphoma**	1 (13%)	1 (9%)	
**Disease Risk Index (EBMT)**			
** Low**	4 (50%)	7 (64%)	0.89
** Intermediate**	4 (50%)	2 (18%)	
** High**	0	2 (18%)	
**Karnofsky score at HCT**			
** <=80%**	3 (38%)	2 (18%)	0.27
** 90%**	3 (38%)	3 (27%)	
** 100%**	2 (25%)	6 (55%)	
**Donor type**			
** Matched sibling**	3 (38%)	4 (36%)	0.71
** Matched unrelated**	4 (50%)	4 (36%)	
** Haploidentical**	1 (12%)	3 (27%)	
**Conditioning**			
** Reduced intensity**	7 (88%)	7 (64%)	0.34
** Myeloablative**	1 (13%)	4 (36%)	
**Patient - Donor sex match**			
** Female into male**	1 (13%)	2 (18%)	1.00
** Other**	7 (88%)	9 (82%)	
**CMV donor/recipient**			
** negative to negative**	2 (25%)	2 (18%)	0.62
** positive to negative**	0	1 (9%)	
** negative to positive**	3 (38%)	2 (18%)	
** positive to positive**	3 (38%)	6 (55%)	
**HCT - comorbidity index**			
** 0**	1 (13%)	3 (27%)	0.45
** 1 or 2**	4 (50%)	4 (36%)	
** >=3**	3 (37%)	4 (36%)	
**Year of HCT**			
** <2018**	3 (38%)	7 (64%)	0.37
** >2017**	5 (62%)	4 (36%)	

ALL, acute lymphoblastic leukemia; AML, acute myeloid leukemia; CML, chronic myeloid leukemia; CMV, cytomegalovirus; EBMT, European Society for Blood and Marrow transplantation; FMT, fecal microbiota transplant; HCT hematopoietic cell transplantation; MDS, myelodysplastic syndrome; MDRO, Multidrug-resistant organisms.

## Results

### Patient Characteristics

Nineteen patients were colonized with an MDRO prior to an allogeneic HCT. These patients were transplanted for hematological malignancies including acute lymphoblastic leukemia (*n*=3), acute myeloid leukemia and advanced myelodysplastic syndrome (*n*=10), chronic myeloid leukemia (*n*=4), T-cell non-Hodgkin lymphoma (*n*=2). HCT was performed using peripheral blood progenitor cells from an HLA matched sibling (*n*=7), unrelated donors (*n*=8) and HLA haploidentical family donors (*n*=4). Myeloablative conditioning was used in 5 patients, while 14 patients received reduced intensity conditioning.

**Figure 1:**
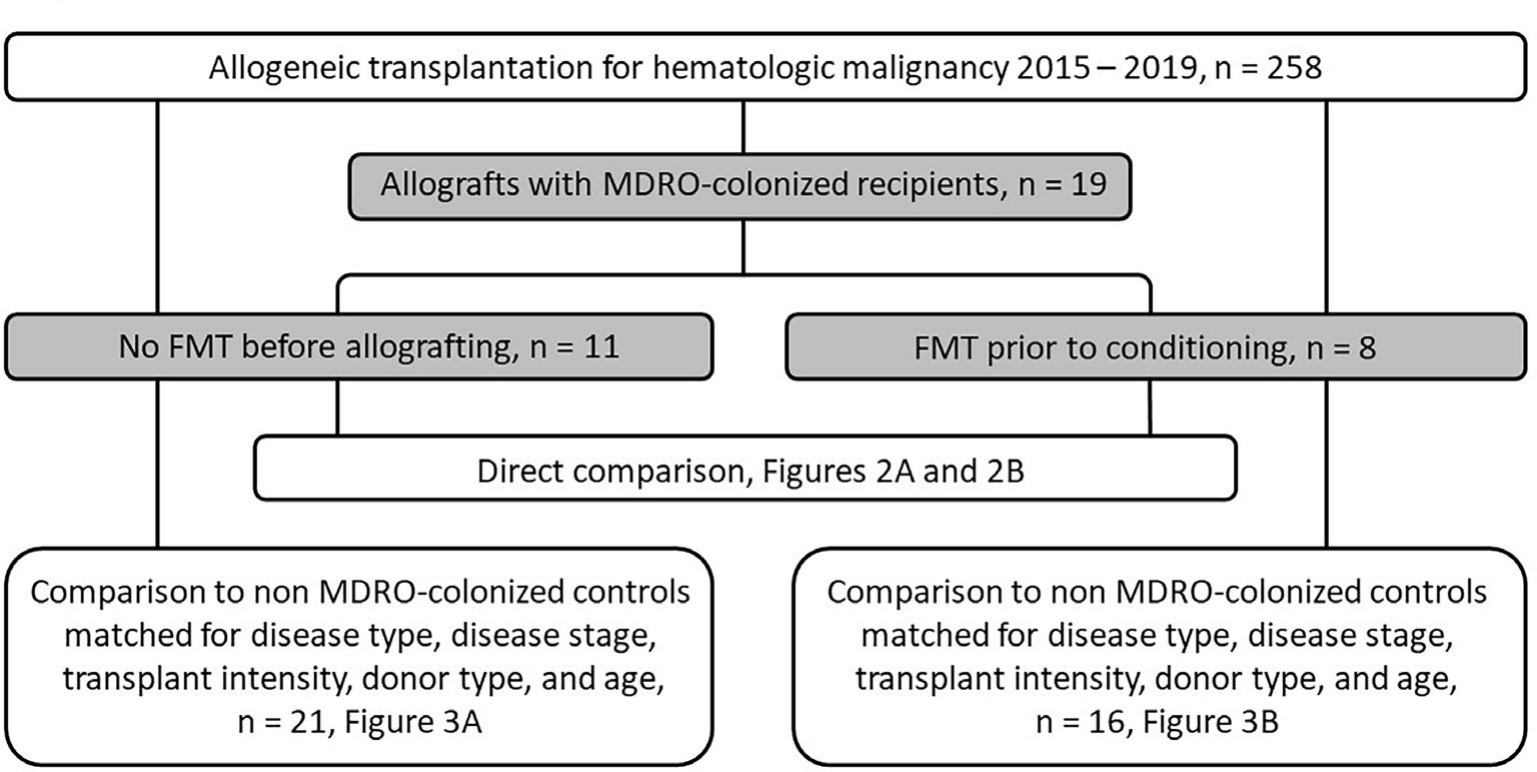
CONSORT diagram.

Eight of the 19 MDRO-colonized patients (‘MDRO-patients’) received FMT (‘FMT MDRO group’); FMT was well-tolerated in all cases, and no adverse events of note were observed that were attributable to the FMT. In seven of these patients, FMT was performed as originally planned 2-6 weeks prior to starting transplant conditioning therapy. None of these patients received antibiotic therapy between the FMT and hematopoietic cell infusion. In one patient, FMT was delayed until three days after the hematopoietic cell infusion due to pre-transplant infection requiring antibiotic therapy. Eleven of the 19 MDRO-colonized patients did not receive FMT (‘No FMT MDRO’ group). Demographics and HCT characteristics of these two groups are provided in **Table 1**, and MDRO characteristics of the patients are listed in **Supplementary Table 1**; also see CONSORT diagram provided in **Figure 1**. Five patients had also previously had MDRO positive blood cultures (four of these in the FMT group) and three in bronchial washings (one in the FMT group) during their treatment prior to HCT.

### Direct Comparison of FMT and No FMT Groups in MDRO Colonized Patients

**Figure 2:**
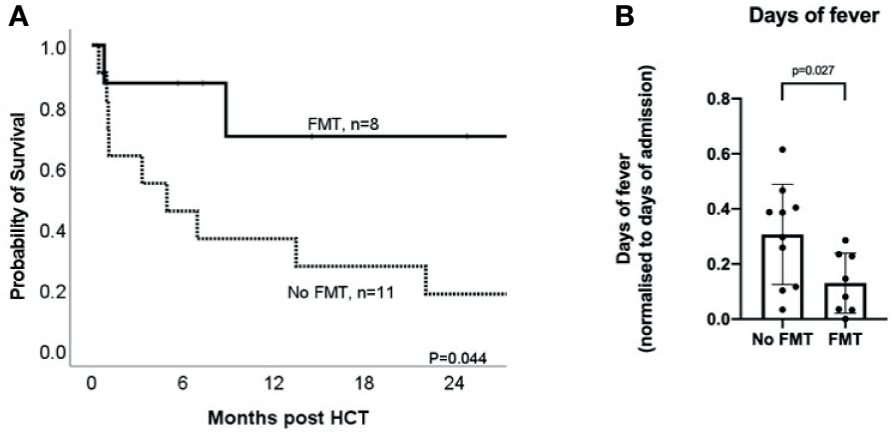
Impact of FMT on clinical outcomes for MDRO-colonized patients receiving HCT. **(A)** Kaplan-Meier curve of overall survival in MDRO-colonized HCT patients who underwent FMT (‘FMT’, solid line; *n* = 8) and those who did not (No FMT, *n* = 11); **(B)** Comparison of days of fever (normalized for days of admission, i.e. number of days with fever divided by total number of admission days) in MDRO colonized HCT patients who underwent FMT (‘FMT’) *vs* those who did not (‘No FMT’).

With a median follow up of 26 months (range 6 – 47 months) for the whole cohort of MDRO-patients, the probability of survival at 12 months post-HCT was 51.0%. The probability of survival of MDRO-patients who underwent FMT was 70% at 12 months, compared to 36% in MDRO-patients who did not (*p*=0.044, **Figure 2A**). Fewer MDRO-patients who underwent FMT needed an admission to the intensive care unit for inotropic support or respiratory failure (0% *versus* 46%, *p=*0.045). MDRO-patients undergoing FMT had fewer days of fever when normalized for the number of admission days (0.11 *versus* 0.29 days, *p=*0.027, **Figure 2B**).

Causes of death were classified as clinical infection in one MDRO-patient in the FMT group, and in five MDRO-patients in the No FMT MDRO group. One additional patient in the No FMT MDRO group died of infection on a background of graft-*versus*-host disease, and one of veno-occlusive disease/sinusoidal obstruction syndrome. One patient in the FMT MDRO group and two in the No FMT MDRO group died of relapse of their malignancy. The one MDRO-patient who died of clinical infection in the FMT MDRO group had no positive blood cultures or other positive bacteriology findings during the two weeks prior to death. Of the five MDRO-patients who died of clinical infection in the No FMT MDRO group, three died with MDRO bloodstream infection, and one with MDRO pneumonia. One died with Candidemia (with no bacterial growth), and one MDRO-patient’s blood cultures were sterile during the two weeks prior to death.

Two of eight (25%) MDRO-patients who underwent FMT achieved MDRO de-colonization; in one MDRO-patient, a new MDRO (different from the original isolate) became detectable. Two of eleven (11%) MDRO-patients in the No FMT MDRO group spontaneously decolonized, and in one MDRO-patient a new MDRO (but not the original) became detectable.

### Matched-Pair Analysis Studies

Even though there were no statistically significant differences in MDRO-patient and HCT characteristics between the FMT and No FMT MDRO groups (**Table 1**), we performed a matched-pair analysis for each group to address the discrepancies in the distribution of some variables between the two groups (e.g. Karnofsky score, conditioning regimen, year of HCT; **Figure 1**). Each of the MDRO-patients who did not receive FMT (‘No FMT MDRO group’) and each of the MDRO-patients who did receive FMT (‘FMT MDRO group’) were matched to two patients without MDRO colonization, but who were matched for the same disease type, disease stage, HCT intensity, donor type (matched sibling, matched unrelated, and haploidentical), and age from our institutional database; for both cohorts, excellent matching was achieved for all included criteria (**Supplementary Tables 2**, **3**). For one patient in the No FMT MDRO group, we could only find one matched control patient rather than two.

**Figure 3:**
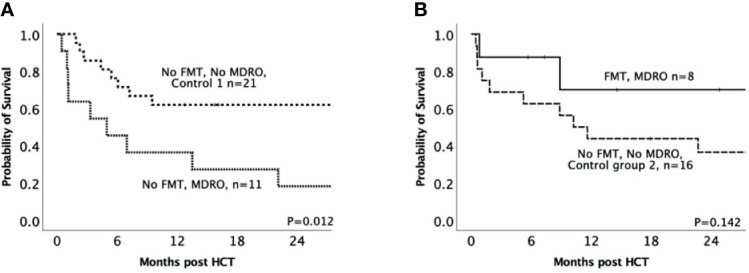
Impact of MDRO colonization on clinical outcomes for patients receiving HCT – matched MDRO *vs* non-MDRO groups. **(A)** Kaplan-Meier curve of overall survival in patients colonized with multidrug-resistant organisms who did not receive FMT (‘no FMT MDRO’, dotted line; *n* = 11) in comparison to controls who were not colonized and were matched for disease type, disease stage, transplant intensity, donor type (matched sibling, matched unrelated, and haploidentical), and age (‘no FMT no MDRO control 1’, dashed line; *n* = 21); **(B)** Kaplan-Meier curve of overall survival in patients colonized with multidrug-resistant organisms who underwent fecal microbiota transplantation (‘FMT MDRO, solid line; *n* = 8) comparison to controls who were not colonized and were matched for disease type, disease stage, transplant intensity, donor type (matched sibling, matched unrelated, and haploidentical), and age (‘no FMT no MDRO control 2’, dash-dotted line *n* = 16).

The No FMT MDRO group had significantly lower survival than their paired cohort not colonized with MDRO (‘no FMT no MDRO control 1’; 36.4% *versus* 61.9% respectively, *p*=0.012, **Figure 3A**) at 12 months. In contrast, there was no statistical difference in survival between the FMT MDRO group and their paired cohort not colonized with MDRO (‘no FMT no MDRO control 2’; 70% *versus* 43.4%, *p*=0.14, **Figure 3B**). Similarly, NRM at 12 months was higher in the No FMT MDRO group (60.2%) than in their matched controls (16.7%, *p*=0.009), but there was no significant difference in NRM between the FMT MDRO group (12.5%) and their matched controls (31.2%, *p*=0.24).

## Discussion

In our non-randomized retrospective analysis and subsequent matched-pair study, FMT mitigated the worse outcomes seen in MDRO-colonized post-HCT patients, including survival. Part of this appears likely attributable to FMT-related impact on infective complications after HCT. For instance, one interesting observation was that whilst most patients in both MDRO groups developed fevers that were treated with antimicrobials as per institutional protocol, there was a clear reduction in the number of days of fever in patients who had received FMT; one interpretation could be that these patients were more responsive to conventional anti-infective treatment. This observation is further supported by the lower requirement for intensive care support in the FMT group, and the lower rate of death from sepsis. Importantly, FMT was also well tolerated in this cohort of immunosuppressed patients, with no serious adverse events; confirming the applicability of its use in immunosuppressed patients (Shogbesan et al., 2018). By performing a matched cohort analysis of those colonized with MDRO, we confirm and extend upon the findings of other groups that MDRO colonization is associated with a poor outcome in the setting of HCT (Bilinski et al., 2016), and, more significantly, describe that the negative impact of MDRO colonization may be ameliorated by pre-HCT FMT. While it has been demonstrated previously that FMT for recurrent *C. difficile* infection is associated with improved overall survival (and reduced BSI) in comparison to antibiotic-treated patients (Ianiro et al., 2019), to our knowledge, this is the first study demonstrating a survival advantage for pre-HCT FMT.

A further noteworthy finding was that the rate of intestinal MDRO eradication after FMT was only 25% in HCT recipients. We have reported previously that the intestinal decolonization rate was 41% in a combined cohort of hematology patients and those with renal insufficiency and recurrent urinary tract infection (Ghani et al., 2020). This rate is similar to both previously published studies of FMT (Battipaglia et al., 2019; Saha et al., 2019), and to the rates of spontaneous decolonization (Davido et al., 2018). As such, our work adds to the mounting evidence that complete intestinal eradication of MDRO appears not to be essential for FMT to mediate clinical benefits in colonized patients.

Although there are apparent clinical benefits observed even without absolute MDRO decolonization, the exact mechanisms by which FMT provides its clinical benefit in MDRO colonized patients are unknown, and more mechanistic studies are required. In mouse models, FMT has been seen to reverse the course of otherwise lethal human bacterial mediated sepsis by enhancing pathobiont clearance *via* the restoration of host immunity in an interferon regulatory factor 3-dependent manner (Kim et al., 2020). As such, part of the benefit of FMT in this setting may involve gut commensal-mediated immune priming, without necessarily significantly suppressing MDRO that colonize the gut (Allegretti et al., 2019; Woodworth et al., 2019; Segal et al., 2020; Bar-Yoseph et al., 2021; Huus et al., 2021). The role that FMT plays in restoring colonization resistance *via* commensal bacteria outcompeting pathobionts for critical nutrients, alteration of pH or oxygen tension, and production of metabolites toxic to them is an another potential contributory mechanism of benefit (Kamada et al., 2013). Supporting this concept, FMT is recognized to restore gut microbial metabolites that are key to gut barrier function (including short chain fatty acids and bile acids) from the low levels found in an antibiotic-exposed gut back to higher levels comparable to healthy stool donors (McDonald et al., 2018; Monaghan et al., 2019; Mullish et al., 2019; Martinez-Gili et al., 2020; Segal et al., 2020). This restoration of gut barrier integrity may limit translocation of pathobionts from the intestinal lumen into the circulation, and therefore reduce the risk of bacteremia. Additionally, aberrant intestinal biodiversity is known to be associated with increased inflammatory response (Belkaid and Hand, 2014) and biomarkers of such an inflammatory response are independent predictors of outcome in HCT, which may in fact be indicative of intestinal inflammation (Artz et al., 2008; Pavlů et al., 2010; Patel et al., 2018).

Our study had certain limitations. The size of our initial cohort was small and non-randomized, with certain discrepancies in clinical characteristics between MDRO-colonized patients who received FMT *versus* those who did not (although none of these reached statistical significance). As such, to add robustness to interpretation, we went on to perform matched pair/case control cohort study with a strict matching strategy in an attempt to minimize any bias. There is a clear indication for further prospective, randomized studies to further investigate the clinical findings here. Further work is also needed to explore the biological explanations for the observed benefit of this therapy, and integrative studies of the interplay of gut microbial profile, their metabolites and gut barrier function are an important next step.

While our experience has been accrued in patients with detectable MDRO in the intestinal microbiome, we hypothesize that these benefits may be more broadly applicable. Due to their previous exposure to broad spectrum antibiotics, chemotherapy, and/or the underlying disease process itself, prospective HCT recipients are recognized to have a relative decrease in diversity of commensal bacteria which are recognized to play a role in immune recovery (Montassier et al., 2015; Andermann et al., 2018). As such, future clinical studies are indicated to explore if FMT may provide comparable clinical benefits even in HCT patients who are not colonized with MDROs.

## Data Availability Statement

The raw data supporting the conclusions of this article will be made available by the authors, without undue reservation.

## Ethics Statement

The studies involving human participants were reviewed and approved by UK Research Ethics Committee (REC reference: 19/LO/0112). The patients/participants provided their written informed consent to participate in this study.

## Author Contributions

BM and RG performed all FMTs. AJI, JA, EO, RP, EK, DM, EB, FD, and JP were involved in selection and care of patients. AI, BHM, RG, RS, JAM, MT, HW, JRM, and JP were involved in the design of this study. AJI, BHM, RG, RS, FD, JRM, and JP analyzed data and wrote manuscript. All authors were involved in review of and critical input into the final manuscript. All authors contributed to the article and approved the submitted version.

## Funding

The Department of Metabolism, Digestion and Reproduction, and the Centre for Haematology at Imperial College London receive funding from the National Institute of Health Research (NIHR) Biomedical Research Centre (BRC) based at Imperial College London and Imperial College Healthcare NHS Trust. BM and AI are the recipients of NIHR Academic Clinical Lectureships, and BM was previously the recipient of a Medical Research Council (MRC) Clinical Research Training Fellowship (grant reference: MR/R000875/1). JA is a NIHR Senior Investigator, FD receives funding from the Medical Research Council (MRC) Clinical Academic Research Partnership Scheme, and JAM was the recipient of a Wellcome Trust Institutional Strategic Support Fund Springboard Fellowship.

## Conflict of Interest

BM reports consultancy fees from Finch Therapeutics Group, outside the submitted work. JRM reports consultancy fees from Enterobiotix Ltd., outside the submitted work.

The remaining authors declare that the research was conducted in the absence of any commercial or financial relationships that could be construed as a potential conflict of interest.

## Publisher’s Note

All claims expressed in this article are solely those of the authors and do not necessarily represent those of their affiliated organizations, or those of the publisher, the editors and the reviewers. Any product that may be evaluated in this article, or claim that may be made by its manufacturer, is not guaranteed or endorsed by the publisher.
