# Supplementary material for: Fecal Microbiota Transplant Mitigates Adverse Outcomes Seen in Patients Colonized With Multidrug-Resistant Organisms Undergoing Allogeneic Hematopoietic Cell Transplantation
Source: Front Cell Infect Microbiol. 2021 Aug 27;11:684659. doi: 10.3389/fcimb.2021.684659 (PMC8430254; doi:10.3389/fcimb.2021.684659)
Supplement: Supplementary file 1 [file DataSheet_1.docx]

***Brief Research Report:***

**Fecal Microbiota Transplant Mitigates Adverse Outcomes Seen in Patients Colonized with Multidrug-Resistant Organisms Undergoing Allogeneic Hematopoietic Cell Transplantation**

**Supplementary Table 1. Details of Multidrug-Resistant Organisms Isolated from HCT recipient patients.**

| **FMT MDRO (n = 8)** | | | **No FMT MDRO (n = 11)** | | |
| --- | --- | --- | --- | --- | --- |
| **Species** | **details** | **N** | **Species** | **details** | **N** |
| **Rectal swab – MDRO screening** | | | | | |
| *Citrobacter freundii* | OXA-48 | 1 | *Citrobacter freundii* | OXA 48 | 2 |
| *Escherichia coli* | ESBL | 2 | *Enterobacter cloacae* | GES-5 | 1 |
|  | IMP-1 | 1 |  | IMP-1 | 1 |
|  | NDM | 1 |  | VIM, IMP-1 | 1 |
| *Klebsiella oxytoca* | GES-5 | 1 | *Escherichia coli* | ESBL | 1 |
| *Klebsiella pneumoniae* | OXA-48 | 2 |  | OXA-48 | 1 |
|  |  |  | *Klebsiella oxytoca* | GES-5 | 1 |
|  |  |  |  | OXA-48 | 1 |
|  |  |  | *Klebsiella pneumoniae* | NDM | 1 |
|  |  |  | *Pseudomonas aeruginosa* | VIM | 1 |
| **Bronchial washing** | | | | | |
| *Klebsiella pneumoniae* | ESBL | 1 | *Enterococcus* spp*.* | van | 1 |
|  |  |  | *Klebsiella oxytoca* | GES-5 | 1 |
| **Blood culture** | | | | | |
| *Escherichia coli* | ESBL | 1 | *Enterococcus* spp*.* | van | 1 |
| *Enterococcus* spp. | van | 2 | *Klebsiella oxytoca* | GES-5 | 1 |
| *Klebsiella pneumoniae* | OXA-48 | 1 |  |  |  |

Abbreviations: ESBL, extended spectrum beta-lactamase; FMT, fecal microbiota transplant; GES, Guiana extended spectrum ESBL; IMP, NDM, OXA, and VIM are types of serine carbapenemases; MDRO, Multidrug-resistant organisms; N, number of patients with a particular microorganism; van, vancomycin resistance.

**Supplementary Table 2. Demographics and further clinical details of No FMT MDRO-colonized HCT recipients and controls**

| **Characteristic** | **No FMT MDRO (n = 11)** | **No FMT No MDRO Control (n = 21)** | ***P*** |
| --- | --- | --- | --- |
| **Patient age (years)       (Median, range)** | 59.8 (31-66) | 51.4 (31-73) | 0.24 |
| **Diagnosis to HCT (years)       (Median, range)** | 0.5 (0.3-13.3) | 0.7 (0.2-21) | 0.82 |
| **Disease**  **CML**  **AML/MDS**  **ALL**  **T-cell lymphoma** | 2 (18%)  7 (64%)  1 (9%)  1 (9%) | 5 (24%)  12 (57%)  2 (10%)  2 (10%) | 0.91 |
| **Disease Risk Index (EBMT)**  **Low**  **Intermediate**  **High** | 7 (64%)  2 (18%)  2 (18%) | 10 (47%)  9 (43%)  2 (9%) | 0.62 |
| **Karnofsky score at HCT**  **<=80%**  **90%**  **100%** | 2 (18%)  3 (27%)  6 (55%) | 2 (10%)  7 (35%)  11 (55%) | 0.89 |
| **Donor type**  **Matched sibling**  **Matched unrelated  Haploidentical** | 4 (36%)  4 (36%)  3 (27%) | 9 (43%)  8 (38%)  4 (19%) | 0.86 |
| **Conditioning**  **Reduced intensity       Myeloablative** | 7 (64%)  4 (36%) | 13 (62%)  8 (38%) | 0.92 |
| **Patient - Donor sex match**  **Female into male**  **Other** | 2 (18%)  9 (82%) | 3 (14%)  18 (86%) | 0.77 |
| **CMV donor/recipient**  **negative to negative positive to negative**  **negative to positive positive to positive** | 2 (18%)  1 (9%)  2 (18%)  6 (55%) | 4 (19%)  4 (19%)  3 (14%)  10 (48%) | 0.89 |
| **HCT - comorbidity index**  **0**  **1 or 2**  **>=3** | 3 (27%)  4 (36%)  4 (36%) | 6 (29%)  10 (48%)  5 (24%) | 0.73 |
| **Year of HCT**  **<2018**  **>2017** | 7 (64%)  4 (36%) | 15 (71%)  6 (29%) | 0.39 |

Abbreviations: ALL, acute lymphoblastic leukemia; AML, acute myeloid leukemia; CML, chronic myeloid leukemia; CMV, cytomegalovirus; EBMT, European Society for Blood and Marrow transplantation; FMT, fecal microbiota transplant; HCT hematopoietic cell transplantation; MDS, myelodysplastic syndrome; MDRO, Multidrug-resistant organisms.

**Supplementary Table 3. Demographics and further clinical details of FMT MDRO-colonized HCT recipients and controls**

| **Characteristic** | **FMT MDRO (n = 8)** | **No FMT No MDRO Control (n = 16)** | ***P*** |
| --- | --- | --- | --- |
| **Patient age (years)       (Median, range)** | 61.9 (33-70) | 59.2 (31-73) | 0.65 |
| **Diagnosis to HCT (years)       (Median, range)** | 0.8 (0.3-9.6) | 0.6 (0.3-13) | 0.82 |
| **Disease**  **CML**  **AML/MDS**  **ALL**  **T-cell lymphoma** | 2 (25%)  3 (38%)  2 (25%)  1 (13%) | 4 (25%)  6 (38%)  4 (25%)  2 (13%) | 1.00 |
| **Disease Risk Index (EBMT)**  **Low**  **Intermediate**  **High** | 4 (50%)  4 (50%)  0 | 8 (50%)  8 (50%)  0 | 1.00 |
| **Karnofsky score at HCT**  **<=80%**  **90%**  **100%** | 3 (38%)  3 (38%)  2 (25%) | 6 (40%)  1 (7%)  8 (53%) | 0.15 |
| **Donor type**  **Matched sibling**  **Matched unrelated  Haploidentical** | 3 (38%)  4 (50%)  1 (12%) | 3 (38%)  4 (50%)  1 (12%) | 1.00 |
| **Conditioning**  **Reduced intensity       Myeloablative** | 7 (88%)  1 (13%) | 12 (75%)  4 (25%) | 0.63 |
| **Patient - Donor sex match**  **Female into male**  **Other** | 1 (13%)  7 (88%) | 2 (13%)  13 (87%) | 0.96 |
| **CMV donor/recipient**  **negative to negative positive to negative**  **negative to positive positive to positive** | 2 (25%)  0  3 (38%)  3 (38%) | 2 (13%)  1 (7%)  6 (40%)  6 (40%) | 0.81 |
| **HCT - comorbidity index**  **0**  **1 or 2**  **>=3** | 1 (13%)  4 (50%)  3 (37%) | 3 (19%)  6 (38%)  7 (44%) | 0.83 |
| **Year of HCT**  **<2018**  **>2017** | 3 (38%)  5 (62%) | 10 (63%)  6 (37%) | 0.39 |

Abbreviations: ALL, acute lymphoblastic leukemia; AML, acute myeloid leukemia; CML, chronic myeloid leukemia; CMV, cytomegalovirus; EBMT, European Society for Blood and Marrow transplantation; FMT, fecal microbiota transplant; HCT hematopoietic cell transplantation; MDS, myelodysplastic syndrome; MDRO, Multidrug-resistant organisms.
